# AMPA GluA1-flip targeted oligonucleotide therapy reduces neonatal seizures and hyperexcitability

**DOI:** 10.1371/journal.pone.0171538

**Published:** 2017-02-08

**Authors:** Nicole M. Lykens, David J. Coughlin, Jyoti M. Reddi, Gordon J. Lutz, Melanie K. Tallent

**Affiliations:** 1 Graduate Program in Pharmacology and Physiology, Drexel University College of Medicine, Philadelphia, Pennsylvania, United States of America; 2 LifeSplice Pharma, Malvern, Pennsylvania, United States of America; 3 Department of Biology, Widener University, Chester, Pennsylvania, United States of America; 4 Department of Pharmacology and Physiology, Drexel University College of Medicine, Philadelphia, Pennsylvania, United States of America; 5 Department of Biochemistry and Molecular Biology, Drexel University College of Medicine, Philadelphia, Pennsylvania, United States of America; University of Modena and Reggio Emilia, ITALY

## Abstract

Glutamate-activated α-amino-3-hydroxy-5-methyl-4-isoxazolepropionic acid receptors (AMPA-Rs) mediate the majority of excitatory neurotransmission in brain and thus are major drug targets for diseases associated with hyperexcitability or neurotoxicity. Due to the critical nature of AMPA-Rs in normal brain function, typical AMPA-R antagonists have deleterious effects on cognition and motor function, highlighting the need for more precise modulators. A dramatic increase in the flip isoform of alternatively spliced AMPA-R GluA1 subunits occurs post-seizure in humans and animal models. GluA1-flip produces higher gain AMPA channels than GluA1-flop, increasing network excitability and seizure susceptibility. Splice modulating oligonucleotides (SMOs) bind to pre-mRNA to influence alternative splicing, a strategy that can be exploited to develop more selective drugs across therapeutic areas. We developed a novel SMO, GR1, which potently and specifically decreased GluA1-flip expression throughout the brain of neonatal mice lasting at least 60 days after single intracerebroventricular injection. GR1 treatment reduced AMPA-R mediated excitatory postsynaptic currents at hippocampal CA1 synapses, without affecting long-term potentiation or long-term depression, cellular models of memory, or impairing GluA1-dependent cognition or motor function in mice. Importantly, GR1 demonstrated anti-seizure properties and reduced post-seizure hyperexcitability in neonatal mice, highlighting its drug candidate potential for treating epilepsies and other neurological diseases involving network hyperexcitability.

## Introduction

Glutamate-activated α-amino-3-hydroxy-5-methyl-4-isoxazolepropionic acid receptors (AMPA-Rs) mediate the majority of brain fast excitatory neurotransmission. The four AMPA-R subunits, GluA1-4, typically assemble as tetrameric cation channels from heterodimers of GluA1, 3 or 4 combined with GluA2, or GluA1 homodimers [reviewed in[1]]. Further GluA subunit diversity is generated by alternative splicing which critically regulates AMPA-R properties including intracellular trafficking, glutamate sensitivity, and desensitization kinetics [2,3]. Ionotropic AMPA-R GluA subunits are alternatively spliced at the flip/flop cassette, and GluA “flip” or “flop” splice variant composition confers substantially different channel properties in the brain [4,5]. Flip and flop are tandem 115 nucleotide cassette exons which code for a portion of transmembrane region 4 and confer only 9–11 amino acid difference between isoforms to regulate channel kinetics and glutamate sensitivity [4]. GluA2-4 flip containing channels have much slower desensitization kinetics with fast resensitization compared to those containing the respective flop isoforms [2,6,7,8]. In contrast, GluA1-flip and GluA1-flop containing channels have similar desensitization kinetics, but GluA1-flip confers greater glutamate sensitivity, giving rise to larger amplitude responses to given concentrations of glutamate than GluA1-flop [4,5]. These characteristics make AMPA-Rs containing GluA-flip isoforms higher gain channels compared to GluA-flop. However, the contributions of GluA flip and flop isoforms to synaptic transmission and neuronal network properties are unknown, due to a lack of experimental compounds that specifically modulate splice variants of individual GluAs.

Dysregulation in the expression of GluA isoforms has been documented in numerous neurological diseases and is thought to be especially critical in diseases involving CNS hyperexcitability and excitotoxicity [9,10], such as epilepsy. Hyperactivation of AMPA-R signaling is also implicated in neurological diseases associated with neurodegeneration and hyperexcitability, making these receptors attractive drug targets [11].Yet no drugs have been developed that target selective GluA subunits, or that regulate GluA alternative splicing.

Despite the large number of antiepileptic drugs (AEDs) that have entered clinical use over the last two decades, approximately 20–30% of patients have seizures resistant to current AEDs. Several studies have shown an increase in GluA1-flip after seizures and in epileptic tissue, in both humans and animal models [12,13,14,15], which is expected to contribute to neuronal network hyperexcitability, seizure, and epileptogenesis. Therefore, a possible strategy to treat epilepsies is to specifically reduce expression of the high-gain GluA1-flip isoform. This highly selective approach should cause fewer adverse effects than non-selective AMPA receptor antagonists, which have dose-limiting effects on brain and motor function. Both AMPA receptor expression [16] and relative flip isoform expression [17] are highest in immature brain, indicating reduction of GluA1-flip may be most effective in treating neonatal and childhood epilepsies, which are particularly refractory to currently available antiepileptic drugs [18,19].

AMPA-R subunits are highly conserved at the protein level, impeding development of conventional small-molecule drugs that selectively modulate receptor isoform composition. However, greater divergence at the genomic level allows selective targeting of specific subunits and isoforms via modulation of pre-mRNA alternative splicing. This can be achieved using splice modulating oligomers (SMOs) designed to sterically block interactions of the spliceosome with splice regulatory sites on pre-mRNA, causing either exon inclusion or exclusion [20,21]. SMOs ameliorate disease symptoms in preclinical models of Duchenne muscular dystrophy (DMD) [20] and spinal muscular atrophy (SMA) [22,23,24], and are in clinical trials for both diseases [25,26].

We have developed a highly selective SMO that regulates splicing of GluA1 pre-mRNA without affecting the splicing of other GluAs. This SMO potently reduced the expression of GluA1-flip transcripts in neonatal mice, yielding a significant reduction in synaptic gain at CA1 hippocampal synapses, protection against seizures, and decreased post-seizure hyperexcitability, with no apparent adverse effect on cognition or motor function.

## Materials and methods

### Experimental design

The objective of the study herein was to develop a compound that could selectively modulate GluA1-flip expression, and determine whether reduction of GluA1-flip expression could be therapeutic as an anti-seizure or anti-epileptic therapy. After developing a candidate drug (GR1), on-target effect and complete specificity to reducing only GluA1-flip expression was determined at the mRNA level and assessed for effect on total GluA1 protein. The effect of GR1 treatment was then characterized at the synapse and network level via whole cell patch clamp and field recordings performed in hippocampal slices, and found to affect changes associated with anti-seizure and antiepileptic properties. Thus, whole animal behavioral experiments were undertaken to investigate anti-seizure properties *in vivo* in a mouse model of chemoconvusant challenge induced seizures. Since many current AEDs produce unwanted side effects on cognition, we also assessed GR1 for potential GluA1-dependent adverse effects on cognition in both cellular and animal models of learning and memory. The GR1 treated, saline-treated, and naïve controls (where applicable) were weight-matched within litter for semi-randomization into treatment groups. Weight matched littermates were also seized at the same time of day in pairs or threesomes to control for diurnal variations in seizure susceptibility across treatment groups. Treatment groups for all seizure and electrophysiology studies were pooled from a minimum of 3 separate litters. Treatment groups for cognitive and motor studies were pooled from a minimum of 2 separate litters.

*Animals* Both male and female FVB mice were obtained by in-house breeding, (FVB/NJ breeding pairs; Jackson Labs; stock number 001800). Pups were weaned between P21 and P25. All animals were housed 1–5 per cage on a 12 h light/dark cycle at 21°C with free access to water and standard diet (Purina 5053—PicoLab Rodent Diet 20). Animals were monitored at least 2x daily and body weights measured daily over the course of dosing and on the day of any seizure or behavioral experiments. During the P12 kainate induced seizure experiments, 60% of the naïve animals reaching status epilepticus (SE) died during seizure. All of the saline-treated or GR1-treated animals survived P12 seizure. All experiments were carried out in strict accordance with the recommendations in the *Guide for the Care and Use of Laboratory Animals* of the National Institutes of Health. As such the early euthanasia/humane endpoint for animals who became severely ill/moribund was loss of greater than 10% body weight in 24 hrs or loss of greater than 20% of peak body weight over the course of the study. No animals were humanely euthanized by this criteria. The animal use protocol was approved by the Drexel University College of Medicine Institutional Animal Care and Use Committee (approval number 17708), and animals were housed in accordance with the *Guide* and Drexel University IACUC guidelines (Drexel University College of Medicine, ULAR facility, Philadelphia, PA). All efforts were made to minimize animal suffering. At the end of study all animals were euthanized by a double kill policy of isoflurane overdose and cervical dislocation.

### SMO design

To develop an SMO that specifically reduced AMPA-R GluA1-flip expression we targeted the 3’ splice site of the GluA1-flip exon, where there is significant sequence divergence between all GluA isoforms. Candidate SMOs were identified which (i) had favorable thermodynamic stability with their complimentary pre-mRNA sequence, (ii) overlapped predicted exonic splice enhancers (ESEs), (iii) avoided predicted exonic splice suppressers (ESSs) and intronic splice suppressers (ISSs), (iv) had 100% identity between mouse and human, and (v) had no significant predicted cross-hybridization to any other genes in the mouse or human genomes.

The RNA Structure program [27] was used to evaluate the predicted open secondary structure of pre-mRNA sequences and the thermodynamic properties of pre-mRNA and SMO interactions. ESE motifs were defined using three prediction tools: ESE Finder [28], RESCUE-ESE [29], and PESX [30]. ESSs were defined using PESX and FAS-ESS tools [31]. Finally, ISEs were predicted using the ACESCAN2 application [32,33]. SMOs were screened using BLASTN analysis for potential hybridization to off-target sites in the human genome. These analyses were combined to identify top candidate SMOs. *In sillico* analysis allowed for *in vivo* target site selection after screening of only 3 candidate SMOs.

### SMOs

All SMOs tested were comprised of 2’-O-methyl bases containing a phosphorothioate substitution on a non-bridging phosphate (TriLink Technologies). Lyophilized SMOs were dissolved in sterile saline (10 μg/μL) and stored at -20°C prior to use or dilution down to dosing concentration. Results for three SMOs are reported here:

GR1-1 (5’-GUUUACGGGACCUCUUCA-3’);

GR1-2 (5’-ACCUUACUUCCGGAGUCCUUGC -3’); and

GR1-3 (5’-GCUAGGUUUACGGGACCUCU-3’).

### ICV injections

Neonatal mice were given single or multiple bilateral ICV injections of either 1 μL 0.9% sterile saline (vehicle control) or 1 μL GR1 dissolved in 0.9% sterile saline per ventricle between postnatal day (P)1-P10 using a custom 33-gauge needle (20° bevel) fit to a 50 μL Hamilton syringe, with coordinates as described previously [23]. Approximate coordinates were as follows: P1: 1 mm caudal from Bregma, 1 mm lateral from the sagittal suture, and 1.5 mm in depth; P3:1–2 mm caudal from Bregma, 1 mm lateral from the sagittal suture, and 1.5 mm in depth; P5 2–3 mm caudal from Bregma, 1–2 mm lateral from the sagittal suture, and 1.5 mm in depth; P10: 3–4 mm caudal to Bregma, 1–3 mm lateral to the sagittal suture, and 2 mm in depth. For clarity, specific injection paradigms are described in the relevant results section or figure legend.

### Real-time PCR

RNA was extracted from freshly dissected samples of cortex and hippocampus using Trizol (Invitrogen) per manufactures instructions, and quantified by 260nm absorbance and evaluated for purity by measuring the 260/280 and 260/230 absorbance ratios (Beckman DU640; Beckman Coulter Inc). Any sample with an absorbance ratio less than 1.8 was excluded. RNA was converted to cDNA (Mulitscribe reverse transcriptase; Applied Biosystems) with random hexamer primers. cDNA (10 ng) was amplified (TaqMan Gene Expressions Master Mix, Applied Biosystems) using a real-time PCR system (MJ Research PT-200 thermalcycler with Chromo4 Real-time PCR Cycler detection block; Bio-Rad). Primers and probes were designed using Primer Express/Filebuilder programs (Applied Biosystems). A common forward primer was selected for amplifying each GluA subunit. See the supplemental figure (S1 Fig) for primer-probe design and relative location of primers and probes within the mRNA transcript.

GluA1 Primers: (Forward: 5’-AAAGGCTATGGCATTGCAACA-3’), (Flip Reverse: 5’-TAAGACGCCTTGCTCACTGAGTT-3’), and (Flop Reverse: 5’ CCCTGCTCGTTCAGTTTTAACA-3’). GluA1 TaqMan MGB Probes: (Flip 5’FAM- CCCTGAGAGGTCCCGTAA-NFQ-3’) and (Flop 5’-FAM-CCCTGAGAAATCCAG-NFQ-3’).

GluA2 Primers: (Forward: 5’-ACGGCATCGCCACACCTA-3’), (Flip Reverse: 5’- TCTAAGACGCCTTGCTCACTGA-3’), and (Flop Reverse: 5’-ACGGCATCGCCACACCTA-3’). GluA2 TaqMan MGB Probes: (Flip 5’FAM-CCTCATTAAGAACCCC-NFQ-3’) and (Flop 5’-FAM-CCTCATTAAGAAATGCGG-NFQ-3’).

GluA3 Primers: (Forward: 5’-TGGTGTGGCAACCCCTAAA-3’), (Flip Reverse: 5’- CACTGAGTTTCAATACTGCAAGGTTT-3’), and (Flop Reverse: 5’- AGAGGCCTTGCTCATTCAGTTT-3’). GluA3 TaqMan MGB Probes: (Flip 5’FAM-TCAGCATTAAGAACGCCT-NFQ-3’) and (Flop 5’-FAM-TAAGAAATGCTGTTAACCTGG-NFQ-3’).

GluA4 Primers: (Forward: 5’-TGGATTCCAAAGGCTATGGTGTA-3’), (Flip Reverse: 5’-AAGACGCCTGCCTCACTGA-3’), and (Flop Reverse: 5’-CCAAGAGGCCTTGTTCATTCA-3’). GluA4 TaqMan MGB Probes: (Flip 5’FAM-TCCTCATTAAGAACTCCTG-NFQ-3’) and (Flop 5’-FAM-TCCTCATTAAGAAATGCTG-NFQ-3’).

β-Actin was used as an endogenous control (Assay ID: Mm00607939_s1, Applied Biosystems). Efficiency of all primer-probe sets were evaluated over 5 logs of cDNA concentration and shown to be equivalent. Real-time PCR reactions were run in duplicate and the level of GluA flip, flop and β-Actin transcripts were adjusted internally by passive reference dye (ROX), quantified by threshold cycle (Ct), and analyzed by the ΔΔCt method [34] using a common threshold setting (500). Fold changes in GluA flip or flop transcript levels for GR1-treated brain regions were calculated relative to saline-treated controls.

### Western blots

40μg of total protein extract from P10 brain tissue homogenate was loaded per well and separated by SDS-PAGE gel (4–15% gradient; Bio-Rad) electrophoresis, and transferred to nitrocellulose membrane. Membranes were blocked with 5% nonfat milk for 1 hr and incubated overnight at 4°C with a rabbit monoclonal GluA1antibody (#ab31212, Abcam, Cambridge, MA) or mouse anti-alpha-tubulin (Sigma) at dilutions of 1:1000 or 1:5000, respectively. Bands were visualized using an immunoperoxidase-conjgated ECL system (SuperSignal West Femto;ThermoScientific) as per manufacturer instructions. Membranes were scanned (Alpha Innotech Imager FluorChem 8900, AlphaEase FC software; FluorChemSP) and integrated intensities of GluA1 and tubulin bands (loading control) were measured using Adobe PhotoShop.

### Seizure studies

All seizure studies were observed and scored in real-time with video recording for documentation and review of results. KA (Ascent Scientific) was dissolved in 0.9% sterile saline (Sigma-Aldrich) and injected IP. Seizure behavior was scored on a modified Racine scale [35], as follows: stage 1, immobility; stage 2, rigid posturing: stage 3, scratching, circling, head bobbing; stage 4, forelimb clonus, rearing, falling; stage 5, severe tonic-clonic behavior; and SE, sustained severe continuous tonic-clonic behavior lasting at least 20 min.

For the single administration seizure model, the KA dose required to elicit seizures without causing death was first established in untreated P10 mice (3 mg/kg), after which saline and GR1-treated mice were tested side by side. Mice were observed and videotaped for 60 min after IP injection of KA and evaluated for highest stage seizure behavior reached during the observation period. Mice typically progressed sequentially through each seizure stage, but occasionally skipped stages.

In the incremental dosing seizure model, for 0.25 and 0.5 μg GR1, we injected 2 mg/kg KA and then 1 mg/kg KA every 20 min until mice reached SE. Due to the much larger doses of KA required to induce SE in mice treated with 1 μg GR1, it was necessary to adjust the protocol for this group and their controls. These mice were initially dosed with 1.5 mg/kg KA and then with 1.5 mg/kg every 20 min until SE was reached. Thus, both groups reached a 3 mg/kg KA dose after their second injection. P10 mice which were given a “second hit” of KA at P12 were given an initial 2mg/kg KA dose followed by 1 mg/kg 20 min later. All of these mice entered SE after the second dose. Mice not reaching SE by 3mg/kg total dose were excluded to control for variances in initial seizure threshold. At 2 h following the beginning of SE, mice were given a bilateral injection of saline or 4 μg GR1. At P12, mice were given 2 mg/kg KA every 20 min until SE was reached. Mice were observed and videotaped until SE was established.

### Hippocampal slice preparation

Hippocampal slices were prepared as previously described [36,37]. Briefly, male or female mice (P10-P12) were anesthetized with isoflurane (4%), decapitated, and the brains rapidly removed and placed in ice-cold glycerol artificial cerebrospinal fluid (ACSF) gassed with 95% O_2_/5% CO_2_ (Carbogen), of the following composition (in mM): 3.5 KCl, 1.25 NaH_2_PO_4_, 5.0 MgCl, 1.0 CaCl_2_, 24 NaHCO_3_, 10 glucose, and 260 glycerol. Slices (300–400 μM) were cut (Vibratome 3000; Vibratome) in glycerol ACSF solution. Hippocampal slices were then incubated at 33°C for 40 min and then room temperature in regular ACSF (in mM: 130 NaCl, 24 NaHCO3, 10 glucose, 3.5 KCl, 1.25 NaH2PO4, 4.0 MgSO4, 4.0 CaCl2), which was used throughout the experiments.

### Whole cell patch clamp recording of CA1 hippocampal neurons

Whole cell patch clamp recordings of hippocampal CA1 neurons were carried out in a submersion recording chamber at room temperature. CA1 neurons were visualized with an Olympus BX51 microscope with infrared transillumination. Pyramidal neurons in the CA1 were identified visually and patched using a glass pipette with tip resistance of 5–7 MΩ. The responses were recorded using an Axon Multiclamp 700B amplifier (Molecular Devices) using PClamp 9.2 software (Molecular Devices) and a 1322A data acquisition board (Molecular Devices). Cells were only included if input resistance was greater than 200 MΩ, the mean being 265 ± 18 MΩ. Recordings in which access resistance changed by more than 15% during the course of the experiment were not included in the analysis. Liquid junction potential was 8 mV and was corrected via the amplifier.

For measurements of EPSC amplitude and sensitivity to cyclothiazide (CTZ), the recording chamber was perfused with normal ACSF augmented with elevated divalent cations (1.5 mM MgSO4 and 2.0 mM CaCl2). Both 10 μM bicuculline and 50 μM D-AP5 were included in the perfusion to block currents generated by GABAA and NMDA glutamate receptors, respectively. The recording pipette contained a gluconate based internal solution (130 mM K-gluconate, 10 mM Hepes, 10 mM glucose, 10 mM KCl, 1 mM EGTA, 0.1 mM CaCl2, 2.0 mM Mg·3ATP, pH 7.3, Osm 290–300). EPSCs were recorded at threshold, maximum and half-maximum stimulation in cells with minimum seal resistance of 200 MΩ. For a subset of cells, 100 μM CTZ was added to the perfusion. EPSCs were recorded every minute for 10–15 minutes following application of CTZ. A response to CTZ typically occurred within 5–10 minutes. Deactivation (τ_m_) was calculated as the weighted mean of the τ_1_ and τ_2_ from a double exponential fit of the deactivation curve from the peak EPSC response [38]. All analysis was done using pClamp 9.2 software. Mean stimulus intensities for saline-treated cells were as follows (in μAmps): threshold 2.4 ± 0.5, half-maximal 4.3 ± 0.6, maximal 6.4 ± 1. In slices from GR1 (2 μg) treated mice, mean stimulus intensities (in μAmps) were 1.9 ± 0.3 for threshold, 4.0 ± 0.6 for half-maximal, and 6.5 ± 8 for maximal. There was no statistical significance in stimulation intensities between the two groups (ANOVA, p > 0.05).

During rectification measurements, the recording chamber was perfused with normal ACSF without augmenting divalent cations but with 10 μM bicuculline and 50 μM D-AP5. The internal solution of the recording pipette for these measurements was cesium based (120 mM CH3O3SCs, 5.0 mM MgCl2, 8.0 mM NaCl, 10 mM EGTA, 10 mM Hepes, 0.5 mM Na3GTP, 2.0 mM Mg·ATP, 0.2 mM 1-naphthyl acetyl spermine, 1 mM QX-314, pH 7.3, Osm 290–300)[39]. The rectification index was calculated as the ratio of EPSC amplitude at Vh = -70 mV over EPSC amplitude at V_h_ = +40 mV. To determine NMDA currents, the recording chamber was perfused as in the rectification experiments, except that D-AP5 was absent. EPSC amplitudes were recorded at a membrane holding voltage of +40 mV, then again after the addition of D-AP5.

### Extracellular recordings

Data was acquired with an Axoclamp 2B amplifier (Molecular Devices) by D/A sampling using pCLAMP acquisition software (Molecular Devices). Extracellular field excitatory postsynaptic potentials (fEPSPs) were recorded at 31°C in the CA1 pyramidal cell layer using a glass micropipette filled with ACSF. Schaffer collateral synaptic responses were evoked at 0.033 Hz with a bipolar tungsten stimulating electrode placed near in the stratum radiatum. Stable baseline fEPSPs were confirmed by stimulating at 40–50% maximal field amplitude for 20–30 min prior to beginning experiments. Baseline fEPSPs were recorded at 40–50% of maximal amplitude for 15 min by stimulating every 30 sec. Train fEPSPs were recorded for an additional 60 min. LTP was generated with two high-frequency trains of 1 s each at 100 Hz, 20 s apart, using the maximal stimulus intensity. We generated LTD using a 15 min 1 Hz train at the half-maximal intensitiy. The mean initial slopes (between the 0 and 50% points on the rising phase) of two averaged fEPSPs were compared between treatment groups.

### Y-maze non-matching to place paradigm

The Y-maze was a Plexiglas constructed of 3 identical arms (internal dimension 33cm x 8cm x 15cm) assembled at 120° angles to each other. Food cups were located in two of the arms; the other arm was used as the “start’ arm. Mice were habituated in the maze on day 1 for 10 min with no food. On subsequent training days, food (0.1 ml of sweetened condensed milk diluted 1:1 with water) was placed in both food cups and the mice were allowed to freely explore and find the food reward, until the mice consistently consumed the food reward and thus were ready for testing. On Testing day food was removed from the home cage for 4 hours before testing. Arms of the Y-maze were baited semi-randomly with condensed milk. Mice were forced into one arm on the first trial by blocking one arm with a plastic block, and on the second trial (15 sec delay) the mouse was rewarded for choosing the un-sampled arm [40]. Six trials per day were run with 10 min inter-trial intervals. Mice reached the criterion for having learned this task when they chose the correct arm at least 75% of the time. In a previous study, control mice readily learn this task, but GluA1 knockouts were impaired such that they never performed above random chance [41]. Thus, this is an uncomplicated, rapidly assayed test for GluA1-dependent cognitive impairment.

### Object recognition memory testing

Novel object recognition testing was performed as previously described [42] with a 1 hour interval between sample and test phases. Briefly, mice were habituated the day prior to testing for 10 min in an empty box (48 x 43 x 38 cm) that was evenly illuminated. The box was cleaned after each trial with 50% ethanol to eliminate any odors and any debris was removed. The following day, mice were placed back in the box with two objects placed 13 cm from either end of the long axis. The objects consisted of two similar wood blocks, approximately 5 x 5 x 5 cm. During a five min interval, the number of times a mouse approached, oriented towards, and sniffed each object was recorded. After a 1 hr delay, the mouse was again placed in the maze with one of the same objects and a novel wooden block that differed in both shape and color. The replacement of objects was random. The mice were given five min to approach the objects. Number of approaches to familiar and novel objects were counted.

### Motor testing

Splay, beam walk, and ladder climb assessments of motor function were performed as described below:

#### Splay

Mice were lifted by the tail to induce a reflexive splaying of the hind limbs. The degree of splay is ranked on a scale of 0 (normal 180°) to 3 (no limb extension), and was performed 2 times.

#### Grip strength

Forelimb grip strength was measured on a Columbus Instruments Computerized Grip Strength Meter (Chatillion Model DFE-002). Mice reflexively grasped the triangular force bar. Maintaining a grip at the base of the tail, mice were pulled by the test administrator with even force at a 180° angle from the force bar until they released their grip. Peak force (N) exerted by the animal on the force bar was measured in triplicate.

#### Beam walk

Mice were placed on a plexiglass beam (4.5” tall X 3/8” thick) and allowed to run along the top. Gait was observed and assigned a number from 0 to 3, depending on the whether the mouse could run normally along the top (0), needed to place one limb on the side for balance (1), needed to place both limbs on the side for balance (2), or could not traverse the beam without falling (3). Ladder Climb: Coordinated locomotion was assessed by the ability of mice to climb up and down a dual-sided ladder. The wire mesh ladder was 16 cm high with an 8 cm platform at the top. Rungs consisted of major (2.5 cm sq) and minor (1.3 cm sq) divisions. Upward and downward climbing angles were 240 and 225 degrees from vertical, respectively. Normal mice are able to traverse the ladder in both directions with ease, gripping all rungs securely without slipping or falling.

### Statistics

Mean data are shown ± standard error of the mean. Statistics were measured using StatistiXL, an add-in program for Microsoft Excel. For all comparisons, significance was defined as p < 0.05. For real-time PCR experiments, the values from saline-treated groups were normalized to 1 and differences between GR1-treated groups was assessed using a one-sample Student’s t-test. Bonferoni correction was applied to p-values for all dose-response data. Unpaired two-sample two-tailed t-tests were used to assess differences between saline and GR1 groups in Western blots, whole-cell patch-clamp experiments, KA dosing after SE, and novel object preference. Two-factor ANOVA was used for extracellular recording data and incremental KA dosing and latency. Proportional analysis of seizure stages and Y-maze performance between saline and GR1-treated groups was performed using Fischer’s exact test. Data is reported as mean ± SEM.

## Results

### The SMO GR1 efficiently and specifically modulates GluA1 splicing

**Fig 1:**
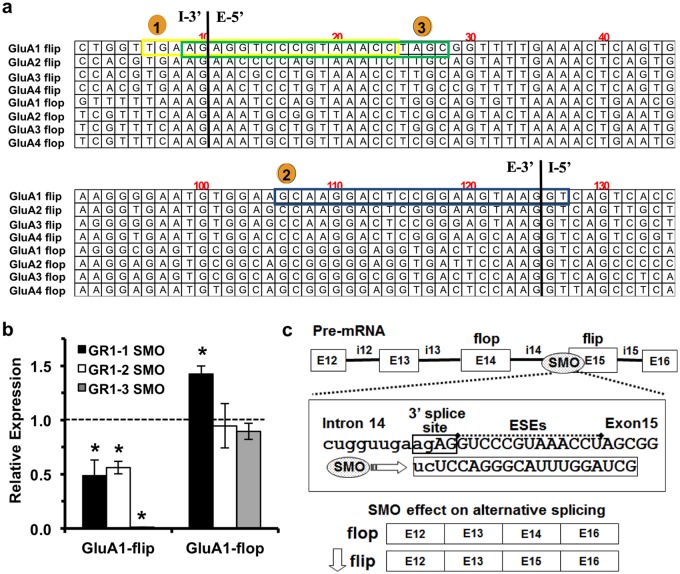
Development of GR1 SMO. **(a)** Target binding location of top three SMO tested for splicing activity against GluA1 flip isoforms; 1. GR1-1 (yellow), 2. GR1-2 (blue), and 3. GR1-3 (green). **(1)** GR1-1 SMO was targeted to the 3’ splice site. **(2)** GR1-2 SMO was targeted to the 5’ splice site. Finally, **(3)** GR1-3 SMO was optimized from the GluR1-1 SMO at the 3’ splice site. Initial in vivo testing was performed in neonatal mice. Mice were injected with SMO (2 μg/ventricle) at postnatal (P) days P1, P3, and P5, tissues harvested at P6, and mRNA expression normalized to saline-injected mice (dotted line). **(b)** Testing of GluA1-flip targeting SMOs in P6 mice (n = 3–5). While all 3 candidate SMOs displayed some on-target splice modulation, only GR1-3 showed a nearly complete knockdown of GluA1-flip with no significant effect on GluA1-flop. Thus, GR1-3 was chosen as the therapeutic SMO to proceed forward in our studies, designated as GR1. (p < 0.05) **(c)** Schematic showing the mechanism of action of GR1 on GluA1 pre-mRNA splicing. The flip and flop exons are tandem 115 nucleotide exons which are spliced from GluA pre-mRNA in a mutually exclusive pattern. Through Watson-Crick base pairing, GR1 binds to the complementary site at the 3’ splice site of the GluA1-flip exon, sterically inhibiting the spliceosome from recognizing the intron/exon boundary and predicted exonic splice enhancer (ESE) motifs, causing exclusion of the GluA1-flip exon.

**Fig 2:**
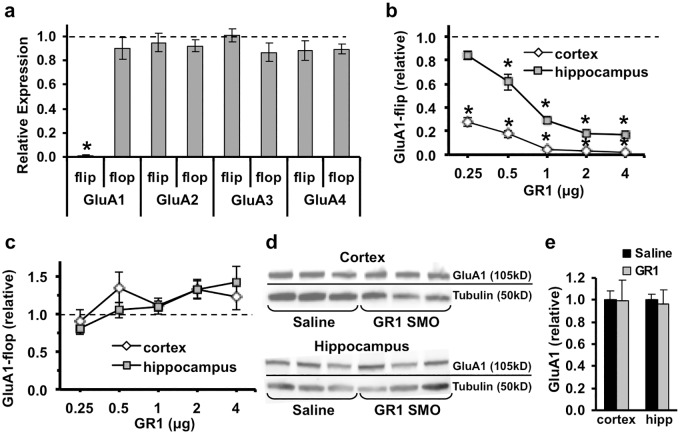
Real-time PCR analysis of GluA isoform expression after ICV delivery of GR1 in neonatal mice. **(a)** Relative expression of GluA1-4 transcripts in brains of P6 mice (n = 5) injected with 2 μg of GR1 at P1, P3, and P5, normalized to saline-injected mice (dotted line). GR1 caused potent and specific reduction of GluA1-flip transcripts (p < 0.001), without affecting any other GluA flip or flop isoforms. **(b)** Dose-response curve of GluA1-flip mRNA expression in hippocampus and cortex of P10 mice after ICV injection of GR1 at P1, P3, and P5 (n = 5–7, p<0.001). **(c)** GluA1-flop mRNA from the same tissue samples as shown in panel **b** exhibited varying levels of concomitant flop upregulation in cortex and hippocampus (p< 0.05). Relative transcript expression was analyzed by one-sample Student’s t-test with bonferoni correction. **(d-e)** GluA1 protein expression in hippocampus and cortex of P10 mice after P1, P3, and P5 ICV injections of saline or GR1 (2 μg per injection). **(d)** Representative GluA1 and tubulin (loading control) Western blots, analyzed from the same blot. **(e)** Mean GluA1 protein expression was not significantly different between GR1 and saline-treated groups in cortex or hippocampus (n = 9 per group; p > 0.05) by unpaired two-sample two-tailed t-test. Asterisks indicate significant difference between GR1 and saline-treated groups.

To develop an SMO that specifically reduces AMPA-R GluA1-flip expression we targeted splice sites flanking the GluA1-flip exon, where there is significant sequence divergence between the various GluA isoforms (Fig 1a). Additional RNA structure and oligonucleotide analysis were used to identify candidate SMOs screened *in silico*. Three top candidate SMOs were delivered to neonatal mouse brains via bilateral intracerebroventricular (ICV) injection and screened for splicing efficiency by rapid (6 day) assay adapted from our previous work [23]. During screening only, SMOs were delivered to neonatal mouse brains using bilateral ICV injections (2 μg per ventricle) on postnatal day 1 (P1), P3, and P5, followed by harvest on P6 and real-time PCR analysis of GluA1-flip expression. The first SMO, GR1-1, masking the 3’ splice site yielded 51.5 ± 14.6% reduction in GluA1-flip (n = 4 GR1-1 and n = 2 saline). Similarly, blocking the 5’ splice site with GR1-2 produced a 43.9 ± 5.9% reduction in GluA1-flip (n = 3 GR1-2 and n = 2 saline). The third candidate SMO (GR1-3) was a product of iterative optimization of GR1-1, and exhibited the desired potency (98.9 ± 0.2% GluA1-flip reduction) and specificity on target (n = 5 GR1-3 and n = 3 saline) as shown (Fig 1b). Hereafter, GR1-3, designated GR1, is exclusively used in this report. The GR1 SMO, and its mechanism of action on pre-mRNA splicing, is depicted in Fig 1c. The target sequence of GR1 is 100% identical between mouse and human, and had no significant cross-hybridization to any other genes in the mouse or human genomes. ICV delivery of GR1 resulted in nearly complete GluA1-flip transcript abolition in brains of P6 mice, and had no significant effect on transcript levels of any other GluA-flip or GluA-flop isoforms (Fig 2a). Because our goal was to evaluate the anti-seizure properties of GR1 in neonatal mice, we measured the dose-response profile of GR1 in P10 mice. Bilateral ICV injections of GR1 on P1, P3, P5, produced a dose-dependent decrease in GluA1-flip transcript levels at P10 in the cortex and hippocampus over a range from 0.25 to 4 μg GR1 (p < 0.001) (Fig 2b). Peak reduction of GluA1-flip transcript was achieved with a GR1 dose of 2 μg per injection, such that GluA1-flip in the cortex and hippocampus was reduced by 97 ± 0.002 and 83 ± 0.02%, respectively, compared to saline-treated controls (Fig 2b). Although there is no statistically significant difference in GluA1-flop expression over the dose range, GluA1-flop trends towards increased expression with increasing doses of GR1 (Fig 2c). Maximal upregulation of GluA1-flop compared to saline-treated controls occurred at the 2 μg dose with increases of 38.3 ± 4.2% in the cortex and 40.4 ± 6.4% in hippocampus (Fig 2c).

GluA1 flip and flop isoforms cannot be distinguished with currently available antibodies. However, Western blots with a GluA1-specific antibody were used to determine how GR1 administration affected total GluA1 protein expression (Fig 2d). Interestingly, ICV delivery of GR1 did not alter GluA1 protein levels in either the cortex or hippocampus compared with saline-treated controls (Fig 2e). These data suggest post-transcriptional regulation functions to maintain GluA1 protein levels constant following nearly complete abolition of GluA1-flip transcripts.

### Directing GluA1 splicing modulates synaptic transmission at CA1 synapses

**Fig 3:**
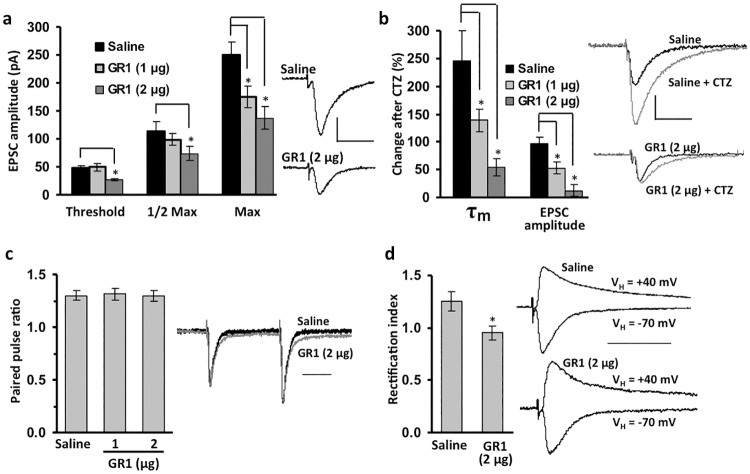
Analysis of AMPA-R mediated EPSCs (aEPSCs) at CA1 synapses in hippocampal slices from P10 mice ICV injected at P1, P3, and P5 with saline or GR1. **(a)** GR1 caused a significant dose-dependent attenuation of aEPSC amplitude versus saline-treated mice (n = 11 per group; p < 0.001). Representative traces of maximal aEPSCs are shown from saline and GR1-treated mice (scale bars: 50 ms; 200 pA). **(b)** Sensitivity to the GluA-flip specific modulator CTZ (100 μM) was significantly reduced in GR1-treated mice compared to saline-treated controls (n = 8–9; p < 0.001). Representative half-maximal aEPSCs are shown from saline and GR1-treated mice before and after CTZ (scale bar: 50 ms, 100 pA). **(c)** Paired-pulse ratio was unchanged after GR1 treatment, relative to saline-treated controls (n = 9 per group; p > 0.05). **(d)** Rectification index of aEPSC amplitude was moderately decreased after SMO treatment (n = 8; p = 0.017). For panels **c** and **d** saline and GR1 representative half-maximal initial aEPSC traces or aEPSCs recorded at -70 mV respectively are normalized to the same amplitude (scale bar: 50 ms). Asterisks indicate significant difference between GR1 and saline-treated groups. Unpaired two-sample two-tailed t-tests were used to assess differences between saline and GR1 groups in whole-cell patch-clamp experiments.

Whole-cell patch clamp was used to establish how GR1-mediated reduction in GluA1-flip modulated AMPA-R mediated evoked excitatory post synaptic currents (aEPSCs) in hippocampal slice preparations from P10 mice. Periodic ICV injections (P1, 3, 5) of GR1 reduced the amplitude of aEPSCs across a range of stimulation intensities (Fig 3a). No significant change in aEPSC decay time (τ_90–10_) was observed between saline (25 ± 1.7 ms) and GR1-treated (28 ± 1.1 ms) groups, respectively (p > 0.05). Sensitivity of aEPSCs to the GluA-flip potentiator cyclothiazide (CTZ) was significantly reduced after GR1 treatment in a dose-dependent manner (Fig 3b), confirming a functional reduction in GluA-flip containing AMPA-Rs at CA1 synapses. These results also suggest GluA1-flip confers much of the CTZ sensitivity to aEPSCs in CA1 and contributes significantly to baseline synaptic transmission. Paired-pulse ratios, a standard measure of presynaptic activity [43], also showed no significant difference between GR1-treated and saline-treated mice (Fig 3c); suggesting that GR1 did not alter glutamate release or presynaptic function. NMDA EPSCs recorded at +40 mV were not significantly different in amplitude between saline (77 ± 16 pA) and GR1-treated (78 ± 15 pA) mice (n = 9; p > 0.05). This further demonstrates GR1 is AMPA-specific and that presynaptic glutamate release was not altered.

The relative amount of Ca^2+^-permeable AMPA-Rs present in an aEPSC can be measured by recording in the presence of internal 1-naphthyl acetyl spermine, a blocker of Ca^2+^-permeable AMPA receptors. The “rectification index” (RI) is the ratio of the amplitude of the aEPSC recorded at -70 and +40 mV. The greater the RI, the larger the proportion of Ca^2+^-permeable AMPA-Rs are present in the aEPSC, whereas an RI of 1 (no rectification) means that the aEPSC contains no Ca^2+^-permeable AMPA-Rs [44]. The RI of aEPSCs recorded at CA1 synapses in slices from saline-treated mice was 1.3 ± .09; in GR1-treated mice the RI was reduced to 1.0 ± 0.07 (Fig 3d; p < 0.05), thus knocking down GluA1-flip appeared to remove all detectable Ca^2+^-permeable AMPA-Rs from the synapse.

**Fig 4:**
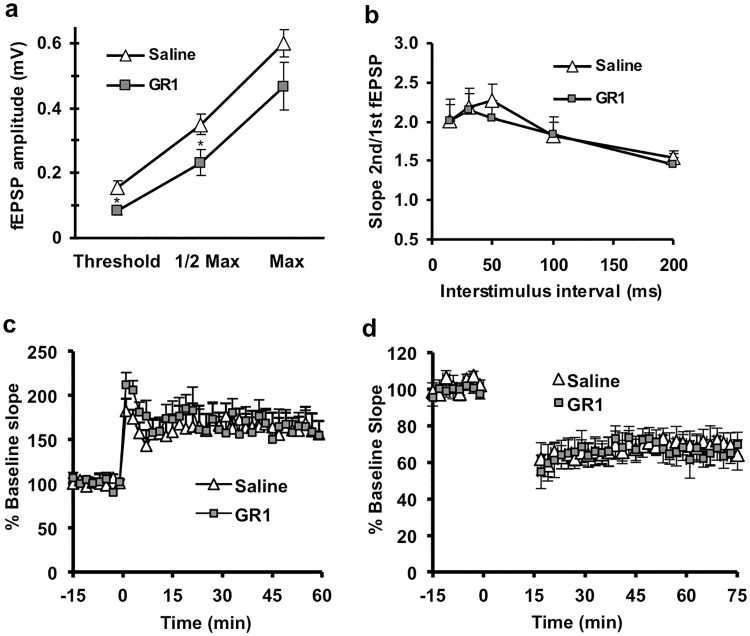
CA1 synaptic function measured extracellularly in P10 hippocampal slices from mice injected at P1, P3, and P5 with saline or GR1 (2 μg). **(a).** Stimulus-response curves for fEPSPs at CA1 synapses showed a reduction in fEPSP amplitude in slices from GR1-treated mice (p < 0.01). **(b)** Paired-pulse facilitation was normal after GR1 treatment (p > 0.05). **(c)** LTP was normal in GR1-treated mice. Data shows % baseline slope before and after two high frequency trains. No difference was observed between slices from saline and GR1-treated mice (p > 0.05). **(d)** LTD was normal in GR1-treated mice. Data shows % baseline slope before and after a 15 min 1 Hz train. No difference was observed between slices from saline and GR1-treated mice (p > 0.05). In all panels **a-c**, n = 7 for saline and GR1-treated groups. In panel **d**, saline n = 8, GR1-treated n = 7. Asterisks indicate significant difference between GR1 and saline-treated groups. Two-factor ANOVA was performed on extracellular recording data.

Trafficking of GluA1 into synapses by high-frequency stimulation underlies many forms of CA1 long-term potentiation (LTP), a kind of synaptic plasticity that models some types of memory and is GluA1-dependent [45]. Therefore, a reduction in GluA1-flip levels could lead to impairment of LTP. Field EPSPs (fEPSPs) at CA1 Schaeffer collateral synapses were significantly reduced in GR1-treated mice (Fig 4a), while paired-pulse facilitation was unchanged (Fig 4b). LTP, measured as percent increase in fEPSP, was unaltered in GR1-treated mice compared to saline-treated mice (Fig 4c), although as expected the amplitudes of the fEPSP were smaller both before and after the trains compared to saline-treated mice. Similarly, long-term depression (LTD) was also unaltered in GR1-treated relative to saline-treated mice (Fig 4d).These results suggest that the remaining GluA1-flop was sufficient to generate both forms of synaptic plasticity.

### GR1 reduces seizure susceptibility and post-seizure hyperexcitability

**Fig 5:**
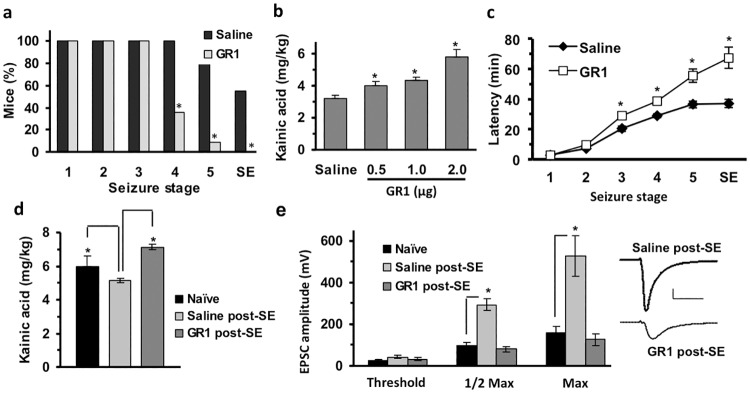
Anti-seizure effects of GR1. GR1 protected neonatal mice from KA-induced seizures and prevented SE-induced increase in aEPSC amplitude. **(a)** Three 2 μg ICV doses of GR1 given at P1, P3, and P5 significantly reduced the percentage of mice progressing to severe seizure stages after a single dose of KA (3 mg/kg- IP) at P10, compared to saline-treated controls (n = 11 per group; p < 0.001 by Fisher’s exact test). **(b-c)** Total KA dose required to elicit SE and latency to reach individual seizures stages at P10 was significantly greater in mice treated with 2 μg (x3 doses) of GR1, compared to saline-treated controls (n = 6 per group; p < 0.05 and p < 0.01 respectively) as analyzed by two-factor ANOVA. **(d)** A single 4 μg dose of GR1, given 2 hours post-P10 SE, prevented the increased susceptibility to “double hit” KA seizure observed for saline-treated SE-experienced mice at P12, compared to naïve controls (*p < 0.05, n = 7 per group). **(e)** Whole-cell patch-clamp recordings of aEPSCs from CA1 pyramidal neurons in P12 mice treated as in panel **d**. Recordings from double hit SE-experience mice demonstrated a large increase in aEPSC amplitude compared to naïve (no SE) mice (p < 0.001) This SE-induced potentiation of aEPSCs was completely prevented by injection of 4 μg GR1 at 2 hr post-SE onset at P10 (n = 5–7 mice per group). Representative traces of maximal aEPSCs are shown for saline and GR1 treated mice post-SE (scale bars: 50 ms, 200 pA). Panels d and e were assessed by unpaired two-tailed t-test.

**Fig 6:**
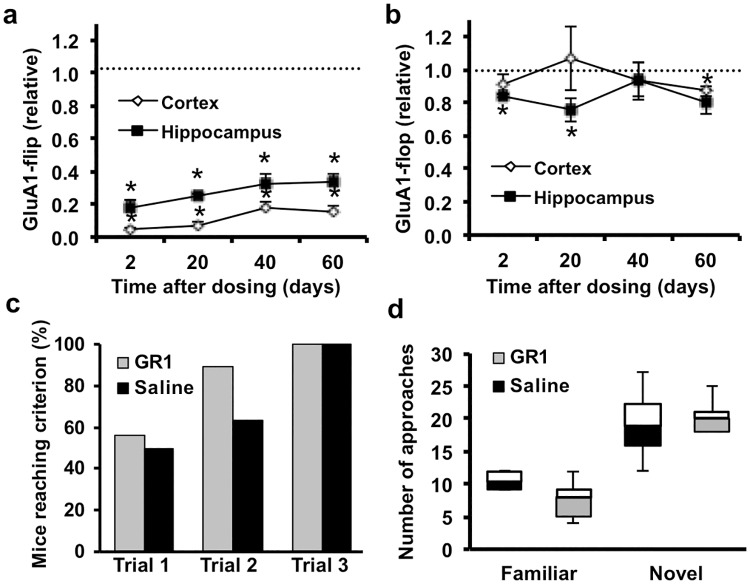
A single ICV injection of GR1 provided a long-lasting reduction in GluA1-flip expression without adverse effects on cognition. Mice were a given ICV injection of GR1 (4 μg) at P10, **(a)** Real-time PCR showed GR1 reduced GluA1-flip in the cortex and hippocampus at all time-points from 2–60 d after administration (n = 4–5; p < 0.001). **(b)** GluA1-flop expression levels, measured in same samples as in panel **a**, were only marginally different between the GR1-treated and saline-treated controls. **(c)** GR1-treated mice did not show impairment in Y maze performance when tested at P30, compared to saline-treated controls (p > 0.05; n = 8–9). **(d)** In the object recognition paradigm, GR1-injected mice showed similar preferences for exploring novel objects as saline-injected controls (p > 0.05 between groups; same cohort of mice as in panel **c**). GluA1-flip expression was analyzed by one-sample Student’s t-test with bonferoni correction, novel object preference by unpaired two-sample two-tailed t-tests, and Y-maze performance by Fischer’s exact test.

We next examined how the GR1-mediated reduction in GluA1-flip affected seizure susceptibility to the chemoconvulsant kainic acid (KA), a glutamate receptor agonist. Kainic acid can be used to assess potential anti-seizure effect in idiopathic neonatal epilepsies, temporal lobe epilepsies of the developing brain [46], and for potential efficacy in several models of severe genetic pediatric epilepsies. For example, mouse models of KCNQ2 mutation [47], SCN8A [48], and SCN1A [49] mutations show an altered threshold to kainate induced seizures. Thus, kainate induced seizures were used to evaluate whether we might have a general anti-seizure effect that could be therapeutically relevant across multiple pediatric epilepsy types. GR1-treated mice were significantly protected from progressing to stage 4–5 seizures and status epilepticus (SE) after KA injection (3 mg/kg; intraperitoneal) compared to saline-treated controls (Fig 5a). An incremental KA dosing regimen also showed GR1-treated mice required more KA to reach SE, compared to saline-treated controls (Fig 5b). Accordingly, latency to reach seizure stages beyond stage 2 was significantly increased in GR1-treated mice, including delayed progression to SE (Fig 5c).To determine whether a single injection of GR1 protected mice against post-SE hyperexcitability, we first evaluated how GluA1-flip and GluA1-flop transcripts were altered at P12 following a single ICV injection of 4 μg GR1 at P10. Single GR1 injections produced an 82 ± 5 and 95 ± 1% reduction in GluA1-flip transcripts in the hippocampus and cortex, respectively (Fig 6a), with no significant effect on GluA1-flop. Incremental doses of KA were used to evoke SE at P10 followed 2 hrs later by injection of GR1 (4 μg) or saline. Mice were re-injected with KA at P12 until SE was observed. In this “double hit” P10 and P12 SE model, mice given 4 μg of GR1 at 2 hrs post-P10 SE required 40% more KA to reach SE at P12 than mice given saline post-SE at P10, and 20% more KA than necessary to induce SE in naïve mice (Fig 5d). In contrast, saline-treated mice showed significantly greater sensitivity to KA compared to naïve mice which did not have KA-induced SE at P10 (Fig 5d) suggesting that post-SE injection of GR1 protected against subsequent SE-induced decrease in seizure thesholds.

This issue was further examined by evaluating if single ICV delivery of GR1 (4 μg– 2μg in 1 μl per ventricle) after induction of SE at P10 reduced post-seizure enhancement of aEPSC amplitude at P12. Similar to other neonatal seizure models [50], the amplitude of aEPSCs was increased more than three-fold following KA-induced SE (Fig 5e). Post-SE injection of GR1 normalized the maximal aEPSC amplitude at P12 to near that of naïve P12 mice (Fig 5e). There was no significant difference between aEPSC amplitudes of naive and post-SE GR1-treated mice (p > 0.05).

To assess the duration of action of GR1, GluA1-flip and flop transcripts were measured in brains at 2–60 days following a single P10 ICV injection of GR1 (Fig 6). Remarkably, 60 d after a single 4 μg injection of GR1, GluA1-flip transcripts were still reduced by 85 and 66% in the cortex and hippocampus, respectively, compared to saline-treated mice (Fig 6a). In the same samples, GluA1-flop transcripts were only marginally altered (Fig 6b).

The sustained activity of GR1 allowed for determination of its potential adverse effects on cognition and motor function. Mice were injected with 4 μg GR1 at P10 and evaluated beginning at P30. Unlike their WT littermates, GluA1 knockout out mice are incapable of learning the Y-maze task, a GluA1-dependent measure of working memory [51,41]. However, ability to learn the Y-maze was not significantly different between GR1 and saline-treated mice (Fig 6c). Similarly, there was no significant difference in novel object recognition between GR1 and saline-treated controls (Fig 6d). No significant differences were found between GR1 and saline-treated mice at P30 for any motor tests including grip strength, splay response, beam walk, and ladder climbing, although by this point GluA1-flip expression had been chronically reduced for 20 days. All GR1 and saline-treated mice showed normal splay reflex, balance beam walk, and ladder climb (score = 0). There was no significant difference in grip strength between the GR1-treated (124.0 ± 4.0 gram-force) and saline-treated (122.6 ± 6.9 gram-force) groups (P = 0.86; unpaired t-test). There was also no significant difference in bodyweight between the GR1-treated (19.4 ± 0.6 g) and saline-treated (19.6 ± 0.6 g) groups (P = 0.80; unpaired t-test).

## Discussion

The SMO developed in this study provided the opportunity to evaluate the impact of direct manipulation of GluA flip/flop isoform expression on AMPA-R mediated synaptic transmission *in vivo*, as a first step to understanding how GluA alternative splicing can be harnessed as an anti-seizure or neuroprotective therapeutic. Compared to AMPA-R’s containing a native mixture of GluA1-flip and flop subunits, our results showed that reducing GluA1-flip levels decreased amplitudes of CA1 aEPSCs, while decay kinetics were unaffected. This suggests GluA1-flip subunits mediate larger synaptic responses than GluA1-flop containing AMPA-Rs. Importantly, these findings confirm *in vitro* studies, that unlike other AMPA-R subunits, flip and flop variants of GluA1 differ in sensitivity to glutamate and resulting synaptic gain, but not in desensitization kinetics [52,53,54]. An increase in GluA1-flip expression, such as occurs in epileptic brain [12,13,14,15], would therefore increase network excitability, leading to increased susceptibility to seizures. The marked decrease in both aEPSCs and CTZ sensitivity seen following a reduction in GluA1-flip expression is also contrary to the current dogma that GluA1 does not contribute significantly to synaptic transmission under basal conditions, but rather is inserted at the synapse only after high-frequency input [55,56]. In support of our results, GluA1 knockout mice showed reduced responsiveness to uncaged glutamate at CA1 synaptic sites [57]. Together this data indicates that GluA1-flip mediates a much larger component of the synaptic response early in development than previously thought, making this particular AMPA-R subunit isoform a prime target to prevent hyperexcitability in neonatal epilepsy.

While a logical target for AEDs, due to the critical nature of AMPA-Rs in normal brain function, non-selective AMPA-R antagonists have many unwanted and sometimes severe side effects. In rodents, AMPA-R antagonists cause sedation, cognitive dysfunction, and motor impairment [58,59,60]. In humans, Talampanel, an AMPA-R antagonist, recently failed clinical trials as an antiepileptic, at least partially due to dose-limiting sedative and motor effects [61,62]. Perampanel, an FDA-approved AMPA-R antagonist antiepileptic, also has unwanted side-effects such as somnolence, dizziness, ataxia, and fatigue [62,63] and carries a “black box” warning for aggression and homicidal ideation and threats (Perampanel package insert, [64]). Drugs that target specific AMPA-R subunits should produce fewer and less severe adverse effects, especially if subtypes that mediate hyperexcitability can be distinguished from subtypes that are required for normal brain function.

LTD expression was not affected by GR1 treatment. Although GluA1 has previously been implicated in LTD [65], several recent reports indicate GluA1 subunits are required for LTP but not LTD [66], and complete loss of AMPA-Rs can be compensated for by kainate receptors to maintain normal LTD responses [67]. Interestingly, decreasing GluA1-flip expression reduced AMPA-R mediated post-synaptic currents in CA1 hippocampus, but did not alter LTP, which underlies specific types of GluA1-dependent memory [41,68]. Likewise, GluA1-depedent learning in the Y-maze paradigm [51,41], was not impaired after GluA1-flip reduction. In a previous study, control mice readily learned this task, but GluA1 knockouts were impaired such that they never performed above random chance [51]. Our behavioral data suggests that selective targeting of GluA1-flip avoids the profound cognitive impairment in spatial working memory found in GluA1 knockout mice, though further testing with a larger sample size will be necessary to determine if milder deficits are present. However, there is a trend toward GR1 treated mice learning the Y-maze task faster than their saline counterparts in early trials, such that a significant deficit in spatial working memory is not likely to emerge with additional testing in this paradigm. Congruent with our LTP and LTD data, after GR1 treatment, the remaining GluA1-flop expression appears sufficient to drive spatial working memory in the Y-maze task. Thus, by using molecular engineering to design a compound that specifically targets GluA1-flip while leaving GluA1-flop and other GluA isoforms intact, we were able to isolate the effects of GluA1 on hyperexcitability from its known role in cognition. Comprehensive analysis of potential adverse effects of GR1 on cognition and motor function in long-term and broader range studies will be required to further validate this finding.

GR1 has other characteristics suggesting it may have a favorable therapeutic profile. Western blot analysis showed that total GluA1 protein levels were not significantly altered after ICV delivery of GR1, even though GluA1-flip transcript levels were reduced by 80–100% and GluA1-flop transcripts were unchanged or modestly increased. This suggests GluA1 flip and flop may be differentially regulated during post-transcriptional processing. Approximately 50% of the total GluA1 mRNA pool generated in the cell soma is trafficked to the dendrites where only a fraction is ultimately translated “onsite” into protein [69]. This suggests the quantity of GluA1-flop mRNA generated after GR1 treatment allows for sufficient mRNA trafficking to the dendrite such that translation into protein is ultimately unchanged but composition is altered to a majority of GluR1-flop subunits. Although, the lack of GluA1-flip and flop specific antibodies does not allow for a direct examination of this issue, our electrophysiological findings support this hypothesis. The 30–40% reduction in amplitude of AMPA-R mediated EPSCs, and greatly reduced sensitivity to the flip-specific potentiator cyclothiazide, can be explained solely by reduced GluA1 flip/flop ratios without a change in overall GluA1 protein levels. Additionally, our finding that the reduction of GluA1-flip by GR1 did not affect LTP or LTD, indicates sufficient GluA1 levels are maintained to allow normal physiological function. Since total GluA1 is not downregulated, compensatory upregulation of other GluA subunits is also unlikely to occur; as demonstrated by our data showing no change in flip or flop transcripts of GluA2-4 after reduction of GluA1-flip by GR1 (Fig 2a).

Because inhibitory interneurons primarily express GluA1-flop [70,71], specific downregulation of GluA1-flip aims to avoid decreasing excitability in interneurons, which would be counterproductive. Thus, GR1 should have less adverse effects than typical AMPA-R antagonists and negative modulators, which act on multiple GluA isoforms. Ca^2+^ permeable AMPA-Rs contribute to seizure susceptibility and seizure-induced neurodegeneration [72,73]. In neonatal hippocampus, Ca^2+^-permeable AMPA-Rs are comprised, at least in part, of GluA1 homomers [74]. Our results show much, if not all, of the Ca^2+^-permeable AMPA-Rs at neonatal CA1 synapses appear to contain GluA1-flip, indicated by the decrease in rectification index of aEPSCs recorded at -70 and +40 mV after reduction of GluA1-flip by GR1. Thus the anti-seizure actions of GR1 are likely mediated, at least in part, by a reduction in Ca^2+^-permeable AMPA-Rs.

Further, we report that the highly soluble GR1 was readily distributed throughout the brain and potently reduced GluA1-flip transcript levels for at least 60 days after a single ICV injection in neonatal mice, and may last much longer. We previously demonstrated that 2’-O-methyl (2’-OMe) SMOs are widely distributed and biologically active throughout the CNS after direct delivery into the CSF [23]. In contrast, another study using the same 2’-OMe SMO at higher concentrations and with more sustained dosing reported no activity in brain [24]. The reasons for the discrepancy between these two studies are unclear. However, the results of the present study validate that SMOs of 2’-OMe chemistry have potent *in vivo* efficacy and extended duration of action on pre-mRNA splicing when delivered directly to the CNS. Importantly, the intrathecally delivered SMO drug, Spinraza (nusinersen), was well tolerated in clinical trials of children [75,76] and infants with SMA [77]. FDA marketing approval for Spinraza was announced December 23, 2016 and establishes the clinical feasibility of intrathecal SMO treatment for neonatal and pediatric patients with serious neurological disorders, such as refractory epilepsy.

Development of new anti-seizure therapies with improved adverse effect profiles may be particularly important for childhood epilepsies. The post-seizure potentiation of AMPA-R containing synapses is thought to be an early event in epileptogenesis, that if reversed, could prevent epilepsy in the developing brain [50]. In support of this hypothesis, GR1-mediated decreases in GluA1-flip expression increased initial seizure thresholds, demonstrating an anti-seizure effect. Further, a single dose of GR1 at 2 hr after SE prevented post-seizure potentiation of aEPSCs and reversed SE-induced decreases in seizure thresholds. If administered closely following a seizure event, GR1 could reduce post-seizure hyperexcitability in neonates. Additionally, an increase in GluA1-flip has been observed in epileptic tissue and post-seizure in humans [12,13] and in several animal models of epilepsy [14,15,78]. Thus, GR1-mediated reduction in GluA1-flip may be an effective strategy for treating patients with diverse epilepsies. Our finding that GR1-treated mice showed delayed progression to early seizure stages, which involve mostly hippocampus, as well as later seizure stages involving cortex, also suggests that reduced GluA1-flip expression could be effective in both partial and generalized epilepsies [79]. However, studies in additional epilepsy models would be needed to confirm these applications for GR1.

Overall, GR1 represents a potential new therapeutic strategy for treating severe neonatal and other epilepsies. Similar SMOs have been developed to abrogate protein defects caused by genetic mutations and two such compounds have recently been approved for treating DMD [25] and SMA [26]. We show here the potential of SMO-mediated control of alternative splice isoform expression from non-mutated genes as a clinical strategy that is applicable to a wide range of neurological disorders.

## Supporting information

S1 Fig Location of primer-probe pairs for all GluA isoforms. Alignment of the last 100 nucleotides (nt) of exon 13 with either the flop (exon 14) or flip (exon 15) exons is shown for GluA1-4. The primer-probe pairs are highlighted for each isoform; GluA1-flop (pink), GluA2-flop (yellow), GluA3-flop (green), GluA4-flop (white), GluA1-flip (orange), GluA2-flip (red), GluA3-flip (blue), and GluA4-flip (black). All probes cross the exon-exon junction between exon 13 and the flip or flop exon. Probes were designed to bind to areas of sequence divergence such that each probe is completely selective for a GluA subunit isoform and in combination with the reverse primer confers single target assay specificity.
(TIF)
