# Supplementary figures and images for: AMPA GluA1-flip targeted oligonucleotide therapy reduces neonatal seizures and hyperexcitability
Source: PLoS One. 2017 Feb 8;12(2):e0171538. doi: 10.1371/journal.pone.0171538 (PMC5298276; doi:10.1371/journal.pone.0171538)

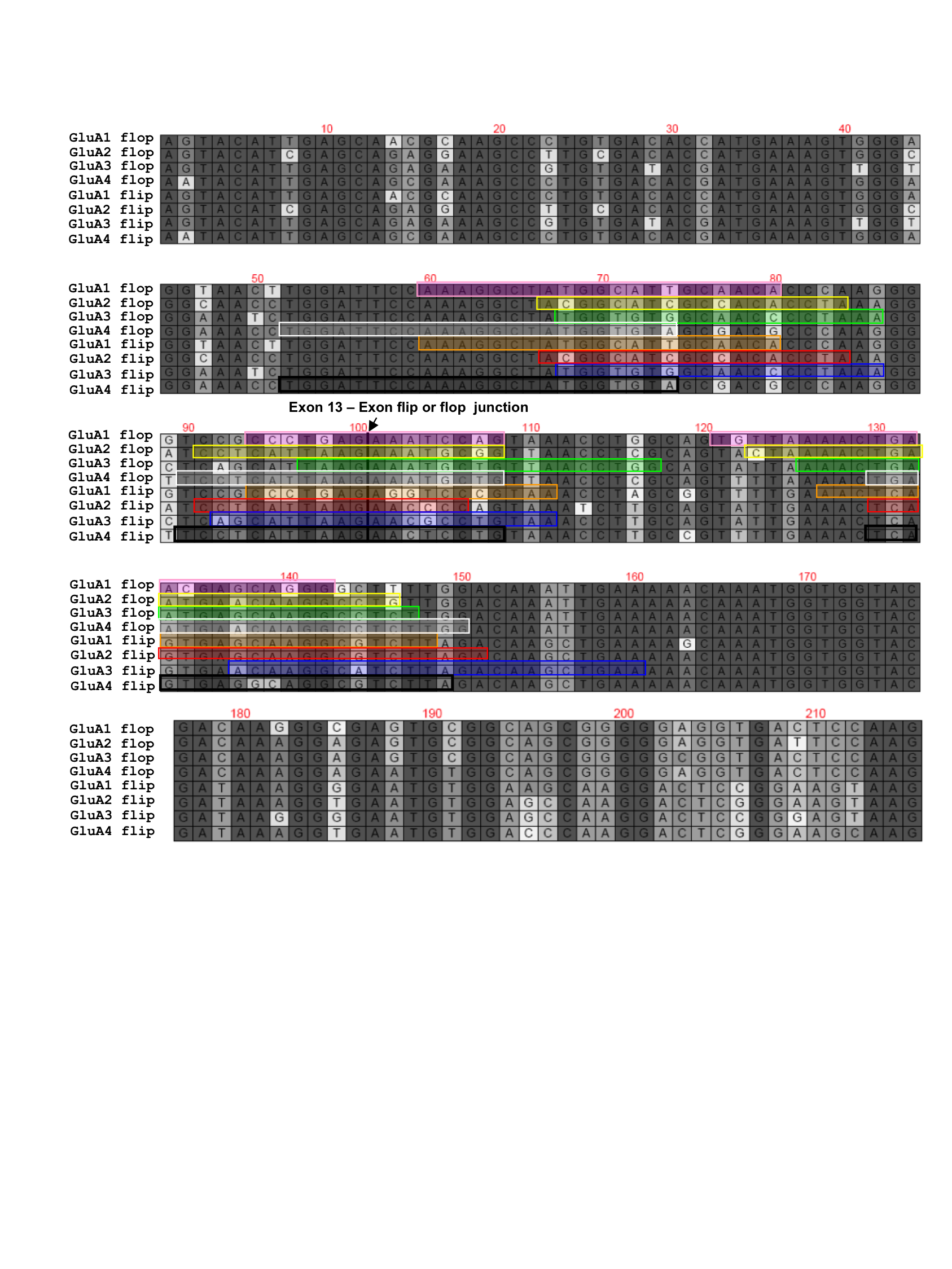

Supplement: S1 Fig — Alignment of the last 100 nucleotides (nt) of exon 13 with either the flop (exon 14) or flip (exon 15) exons is shown for GluA1-4. The primer-probe pairs are highlighted for each isoform; GluA1-flop (pink), GluA2-flop (yellow), GluA3-flop (green), GluA4-flop (white), GluA1-flip (orange), GluA2-flip (red), GluA3-flip (blue), and GluA4-flip (black). All probes cross the exon-exon junction between exon 13 and the flip or flop exon. Probes were designed to bind to areas of sequence divergence such that each probe is completely selective for a GluA subunit isoform and in combination with the reverse primer confers single target assay specificity. (TIF) [file pone.0171538.s001.tif]
